# Early life antibiotic-use and risk of childhood type 1 diabetes: a nationwide cohort study utilising data linkage

**DOI:** 10.48130/pedi-0026-0003

**Published:** 2026-06-24

**Authors:** Sharan Ram, Marine Corbin, Amanda Eng, Amanda Kvalsvig, Michael G. Baker, Andrea 't Mannetje, Anna Davies, Jeroen Douwes

**Affiliations:** 1 Centre for Public Health Research, Massey University, Wellington, 6021, New Zealand; 2 Department of Public Health, University of Otago, Wellington, 6021, New Zealand

**Keywords:** Antibiotics, Early-life, Childhood, Prenatal, Postnatal exposure, Type 1 diabetes, Data-linkage

## Abstract

Antibiotic use in early life is associated with autoimmune diseases like Type 1 diabetes (T1D), but evidence is inconsistent. We examined associations of prenatal and early-childhood (0–5 years) antibiotic use and T1D in New Zealand, a country with high antibiotic consumption. Using linked data, we identified antibiotic use during pregnancy and the first 5 years for all children born between 2005 and 2010 (*n* = 315,789), followed until 2021. Associations were assessed using Cox proportional hazard regression, adjusted for parental and perinatal factors. For prenatal exposure, we assessed associations with T1D-diagnosis from birth; for early-childhood exposure, associations were assessed with T1D-diagnosis from age 5. Antibiotic use (≥ 1 course) was 29.2% for pregnant mothers and 96.3% for children aged 0–5 years. T1D was diagnosed in 0.36% (*n* = 1,131), with 0.28% (*n* = 906) diagnosed after age 5 years. Antibiotic use during pregnancy was associated with T1D, both expressed as a continuous variable (HR 1.05, 95% CI 1.03–1.08) and as a dichotomous variable (0 vs ≥ 1 course; HR 1.20, 1.05–1.38). When categorised into 0, ≤ 1, ≤ 2, and ≥ 3 courses, a significant (*p* = 0.0008) positive trend was observed. For postnatal exposure, the HR was 1.02 (1.02–1.03) and 1.62 (1.00–2.63), respectively; when categorised into 0, ≤ 4, ≤ 7, ≤ 12, and ≥ 13 courses, a significant dose-response trend (*p* = 0.0017) was shown. Strongest associations were found for moderate and broad-spectrum antibiotics in both periods. Prenatal penicillin and cephalosporins/cephamycins-use, and postnatal exposure to all antibiotic classes except tetracyclines were associated with a higher risk of T1D. In conclusion, antibiotic use during both prenatal and early childhood is associated with an increased risk of T1D.

## Introduction

Type 1 diabetes (T1D) is one of the most common autoimmune disorders in young people. The incidence varies across age groups and regions, with the highest incidence in those aged 10–14 years (18.02/100,000) and the lowest in 15–19-year-olds (6.71/100,000)^[1]^. The incidence has increased over the past few decades^[2,3]^, and simulations project a further global increase in the next 30 years^[4]^.

Although the aetiology of T1D is still largely unclear, it is widely accepted that it is a complex autoimmune disorder influenced by multiple factors, including genetic predisposition, immune system dysregulation, and environmental triggers. While genetic factors play a crucial role, the rising incidence of T1D suggests that environmental factors may increasingly contribute to disease development^[5]^.

There is evidence that the gut microbiome may be involved in T1D^[6]^, with animal models showing that alterations in the intestinal microbiota increase the risk of T1D^[7,8]^. Studies in humans have also found marked differences in composition and diversity of the gut microbiota in T1D patients^[9,10]^. However, due to the cross-sectional nature of these studies, it cannot be excluded that this association is the result of the disease process itself and/or related treatment.

Several studies have assessed associations with pre-^[11−14]^ and post-natal antibiotic use, with some reporting positive and dose-dependent associations^[13,15,16]^ and others finding no association^[11,14,17−20]^. Mixed results may be explained by reliance on self-reported antibiotic use, small sample sizes, and sometimes short follow-up; if causal, positive associations may be explained by antibiotic-associated microbiota dysbiosis. This results in an imbalance of beneficial and harmful bacteria, which may, particularly during the critical early-life period when the infant microbiome is rapidly developing, disrupt immune maturation, homeostasis, and subsequent risk of immune or autoimmune-related conditions such as T1D^[6,21−23]^. The extent of these effects depends on the spectrum and timing of antibiotic exposure, with broad-spectrum agents producing the most persistent microbial shifts^[22−26]^.

Most prior studies on antibiotic use and T1D risk have been conducted in countries with relatively low use of antibiotics, unlike New Zealand, which ranks amongst the highest antibiotic users in the OECD^[27]^. Furthermore, earlier research has often relied on self-reported exposures and limited follow-up, which can introduce bias and limit generalisability. In contrast, we conducted a nationwide cohort study using linked data (reducing the risk of recall bias) to assess associations between antibiotic use in early life (both *in utero* and the first 5 years) and the risk of T1D in all children born in New Zealand between 2005 and 2010 and followed until 2021. We assessed associations for different classes of antibiotics and whether caesarean section (CS, known to affect the gut microbiota^[28,29]^) affects these associations. We hypothesise that antibiotic use in early life is associated with an increased risk of childhood T1D.

## Design and methods

### Study population

The cohort consisted of all children born in New Zealand between October 2005 and December 2010 (*n* = 315,789) identified from the Department of Internal Affairs (DIA) births registry within Statistics New Zealand’s (Stats NZ) Integrated Data Infrastructure (IDI). The IDI is a longitudinal meta-dataset of routine datasets administered by government agencies, Stats NZ surveys (including the 2018 census), and non-government organisations^[30,31]^. Children who were stillborn, died before their fifth birthday, or migrated overseas before the age of 5 years were excluded, while all singleton and non-singleton births were included. Children were followed from the end of the antibiotic exposure periods (see below) until T1D diagnosis, death, emigration, or until the end of 2021 (age 11–16 years). Prenatal antibiotic use was assessed through the mothers who were identified from the DIA births registry. A detailed flow diagram illustrating cohort inclusion, exclusions, and final sample size is presented in Supplementary Fig. S1.

This study is part of a larger study, described in a previously published protocol paper, which provides the methods of the overall research in detail^[32]^.

### Antibiotics exposure

Exposure to antibiotics *in utero* (measured by the number of antibiotic courses dispensed to the mother during pregnancy) and antibiotics dispensed to the child for different postnatal periods (≤ 2 years and ≤ 5 years) were identified and counted for all cohort members from pharmaceutical data. The postnatal periods selected represent critical windows during early life when antibiotics may have the greatest impact on the developing microbiome^[33]^. Each antibiotic prescription was categorised by: (1) class, according to the Anatomical Therapeutic Chemical (ATC) classification J01 'Antibiotics for systemic use' (e.g., penicillins, cephalosporins); and (2) spectrum (broad, moderate, or narrow)^[32,34]^. A detailed breakdown of antibiotic classes, sub-classes, and their assigned spectrum is provided in Supplementary Table S1.

### T1D ascertainment

T1D was determined by linking the cohort with the following data collections in the IDI: (1) hospital discharges; (2) pharmaceutical prescriptions of insulin; (3) laboratory claims data (data on primary care testing subsidies); and (4) National Non-Admitted Patient Collection (outpatient data with diagnosis indicated in the purchase unit). We used three algorithms: the first is based on integrating T1D prescriptions and T1D hospitalisation data, the second is based on the McKergow algorithm^[35]^, and the third is based on the New Zealand 'Health Tracker'^[36]^. Comprehensive details regarding each algorithm are provided in Supplementary Table S2. The date of the first insulin prescription or hospital admission for T1D was used to determine the year of diagnosis.

### Confounders and other covariates

Fixed covariates/confounders included: sex (male/female); prioritised ethnicity (Māori, Pacific, Asian, Middle Eastern, Latin American & African, Others, and European)^[37]^; 2018 NZ Deprivation Index (NZDep)^[38]^, a census-based index with a relative deprivation score assigned to each geographical mesh block of residence (1–10, with 10 representing highest deprivation); rurality (urban/rural)^[39]^; birth weight (low, normal, high, only applied for postnatal exposure models); gestation (preterm, term, post-term, late-term); mode of obstetric delivery (vaginal delivery, emergency CS, and planned CS); and maternal age at childbirth.

### Statistical analysis

Analyses were conducted using SAS, v9.4 (SAS Institute Inc., Cary, NC). We used Cox proportional hazards regression to assess associations with T1D, with adjustment for potential confounders; for key covariates (sex, ethnicity, deprivation, mode of obstetric delivery), we also conducted stratified analyses and tested for interaction. Risk estimates are presented as hazard ratios (HRs) with 95% confidence intervals (CIs). Analyses are presented using the first T1D algorithm, with analyses repeated for the other two algorithms. Follow-up commenced after the completion of each exposure window (from birth for prenatal exposure and from age 5 years for early-childhood exposure). A washout period was also applied, excluding T1D diagnoses occurring within the first 6 months after the exposure window to minimise the risk of reverse causation bias. To further explore temporal patterns of risk, age-specific Cox models were used; i.e., prenatal antibiotic exposure was examined across three follow-up periods (birth to < 5, ≥ 5 to < 10, and ≥ 10 years), and postnatal exposure across two periods (≥ 5 to < 10, and ≥ 10 years). Children diagnosed with T1D in earlier periods were excluded from subsequent analyses.

For prenatal antibiotic use, we used both a continuous (based on the number of courses of antibiotics prescribed) and a categorical variable (i.e., ever/never and 0, ≤ 1, ≤ 2, ≥ 3 courses of antibiotics) with categories based on the 25^th^ percentiles. We repeated the ever/never analyses for specific subclasses of antibiotics (penicillin, macrolides, cephalosporins and cephamycins, tetracyclines, 'other' antibiotics), and for spectrum (broad, moderate, narrow).

For postnatal antibiotic use, associations were assessed using different time windows of antibiotic use, i.e., ≤ 5 years and ≤ 2 years. We conducted analyses using antibiotic use, both as a continuous and a categorical variable (i.e., ever/never and 0, ≤ 4, ≤ 7, ≤ 12, and ≥ 13 prescriptions or 0, ≤ 3, ≤ 6, ≤ 9, ≤ 14, and ≥ 15, depending on the specific time window applied—see results), using both 20^th^ and 25^th^ percentiles. We repeated the ever/never analyses for specific subclasses of antibiotics and for spectrum (see above).

Trend analysis for dose-response associations using different antibiotic-prescription categories was assessed using the Cox regression model. To test whether associations differed for children born by CS, we stratified the analysis by mode of obstetric delivery, using antibiotic exposure as a continuous variable.

In compliance with the IDI confidentiality requirements, all frequencies/counts were rounded to the nearest multiple of three, and percentages were calculated from the rounded counts (hence the number of total participants in each table varying slightly, and percentages not adding up to exactly 100%). All statistical tests were performed on unrounded counts. All counts under six and the HRs derived from these are suppressed according to IDI confidentiality requirements (marked as 'S' in the tables).

## Results

A total of 315,789 children were included, with population characteristics presented in Table 1.

**Table 1:** Characteristics of the study population for children born in New Zealand between 1 October 2005 and 30 December 2010 stratified by antibiotic exposure status during gestation and ages 0–5 years (exposed vs unexposed).

Variables		Gestation				0–5 years		
		Unexposed (%)	Exposed (%)	Total (%)		Unexposed (%)	Unexposed (%)	Total (%)
Sample size		222,099	93,687	315,789		11,718	303,846	315,567
Sex	Male	114,216 (51.4)	48,351 (51.6)	162,567 (51.5)		5,607 (47.9)	15,6843 (51.6)	162,450 (51.5)
	Female	107,883 (48.6)	45,339 (48.4)	153,219 (48.5)		6,108 (52.1)	147,003 (48.4)	153,111 (48.5)
Ethnicity	NZ Europeans	108,834 (49)	35,475 (37.9)	144,309 (45.7)		6,255 (53.4)	137,904 (45.4)	144,156 (45.7)
	Māori	62,733 (28.3)	35,811 (38.2)	98,544 (31.2)		2,478 (21.3)	96,021 (31.6)	98,499 (31.2)
	Pacifica	21,036 (9.5)	12,981 (13.9)	34,017 (10.8)		921 (7.9)	33,084 (10.9)	34,005 (10.8)
	Asian	23,271 (10.5)	7,272 (7.8)	30,543 (9.7)		1,641 (14)	28,893 (9.5)	30,534 (9.7)
	Middle Eastern, Latin American, and African (MELAA)	2,724 (1.2)	1,095 (1.2)	3,816 (1.2)		228 (1.9)	3,588 (1.2)	3,813 (1.2)
	Others	3,501 (1.6)	1,059 (1.1)	4,563 (1.4)		195 (1.7)	4,359 (1.4)	4,557 (1.4)
Deprivation Index deciles	10 (most deprived)	30,360 (13.7)	20,352 (21.7)	50,712 (16.1)		1,134 (9.7)	49,563 (16.3)	50,694 (16.1)
	9	24,813 (11.2)	13,494 (14.4)	38,304 (12.1)		1,140 (9.7)	37,137 (12.2)	38,274 (12.1)
	8	22,224 (10)	10,569 (11.3)	32,793 (10.4)		1,083 (9.2)	31,680 (10.4)	32,763 (10.4)
	7	21,483 (9.7)	9,009 (9.6)	30,492 (9.7)		1,122 (9.6)	29,352 (9.7)	30,474 (9.7)
	6	20,340 (9.2)	7,617 (8.1)	27,957 (8.9)		1,020 (8.7)	26,910 (8.9)	27,930 (8.9)
	5	19,716 (8.9)	6,750 (7.2)	26,466 (8.4)		1,137 (9.7)	25,305 (8.3)	26,439 (8.4)
	4	18,810 (8.5)	6,177 (6.6)	24,993 (7.9)		1,053 (9.0)	23,919 (7.9)	24,975 (7.9)
	3	17,601 (7.9)	5,226 (5.6)	22,830 (7.2)		981 (8.4)	21,828 (7.2)	22,806 (7.2)
	2	16,527 (7.4)	4,704 (5.0)	21,231 (6.7)		1,011 (8.6)	20,205 (6.7)	21,216 (6.7)
	1 (least deprived)	13,623 (6.1)	3,786 (4.0)	17,409 (5.5)		774 (6.6)	16,623 (5.5)	17,394 (5.5)
Rurality	Urban	171,879 (77.4)	73,761 (78.7)	245,640 (77.8)		8,436 (72.0)	237,039 (78.0)	245,475 (77.8)
	Rural	33,642 (15.2)	13,926 (14.9)	47,568 (15.1)		2,019 (17.2)	45,504 (14.9)	47,526 (15.1)
	Missing	16,581 (7.5)	6,000 (6.4)	22,581 (7.2)		1,263 (10.8)	21,303 (7.0)	22,566 (7.2)
Birthweight	Low birth weight (LBW) (< 2,500 g)	11,475 (5.2)	6,774 (7.2)	18,249 (5.8)		642 (5.5)	17,595 (5.8)	18,237 (5.8)
	Normal birth weight (NBW) (2,500–4,000 g)	174,972 (78.8)	72,816 (77.7)	247,785 (78.5)		9,225 (78.7)	238,380 (78.5)	247,605 (78.5)
	High birth weight (HBW) (> 4,000 g)	34,863 (15.7)	13,809 (14.7)	48,672 (15.4)		1,764 (15.1)	46,881 (15.4)	48,642 (15.4)
Type of delivery	Caesarean section (elective)	22,512 (10.1)	10,539 (11.3)	33,051 (10.5)		1,119 (9.6)	31,908 (10.5)	33,027 (10.5)
	Caesarean section (emergency)	29,934 (13.5)	12,711 (13.6)	42,645 (13.5)		1,296 (11.1)	41,316 (13.6)	42,612 (13.5)
	Non-caesarean section	162,555 (73.2)	67,560 (72.1)	230,118 (72.9)		8,049 (68.7)	221,904 (73.0)	229,953 (72.8)
	Missing	6,765 (3.1)	2,748 (2.9)	9,513 (3.0)		1,242 (10.6)	8,262 (2.7)	9,507 (3.0)
Gestational age	Full-term (37 weeks ≤ gestational age < 40 weeks)	119,202 (53.7)	48,363 (51.6)	167,568 (53.1)		6,318 (53.9)	161,145 (53.0)	167,463 (53.1)
	Pre-term (gestational age < 37 weeks)	52,644 (23.7)	27,288 (29.1)	79,932 (25.3)		2,685 (22.1)	77,175 (25.4)	79,857 (25.3)
	Late-term (41 weeks ≤ gestational age < 42 weeks)	40,989 (18.5)	14,703 (15.7)	55,689 (17.6)		2,163 (18.5)	53,493 (17.6)	55,653 (17.6)
	Post-term (≥ 42 weeks)	9,141 (4.1)	3,300 (3.5)	12,444 (3.9)		495 (4.2)	11,943 (3.9)	12,438 (3.9)
Maternal age at delivery (years)	< 25	45,039 (20.3)	30,030 (32.1)	75,069 (23.8)		1,935 (16.5)	73,089 (24.1)	75,027 (23.8)
	< 30	52,344 (23.6)	22,077 (23.6)	74,418 (23.6)		2,754 (23.5)	71,613 (23.6)	74,364 (23.6)
	< 35	67,329 (30.3)	22,221 (23.7)	89,553 (28.4)		3,585 (30.6)	85,911 (28.3)	89,496 (28.4)
	≥ 35	57,375 (25.8)	19,359 (20.7)	76,734 (24.3)		3,447 (29.4)	73,221 (24.1)	76,668 (24.3)
Counts are randomly rounded to comply with Statistics New Zealand’s data privacy requirements; as a result, totals may be slightly different across different variables and analyses.								

### Prenatal antibiotics

In total, 29.2% of mothers were prescribed antibiotics during pregnancy. The median follow-up was 13.5 years from birth (range 0–17 years), with 0.36% (*n* = 1,131) children diagnosed with T1D during this period using the first algorithm (see Methods). The mean age at diagnosis was 9.0 years (range 0–17).

When antibiotic use was analysed as a continuous variable, each additional prescription was associated with a 5% increase in risk (*p* < 0.05; Table 2). A significantly increased risk was also shown when we compared ever vs never antibiotic use (HR 1.20, 95% CI 1.05–1.38), and a significant dose-response trend (*p* = 0.0008) was observed when comparing different categories of antibiotic use.

**Table 2:** Association between antibiotic use during pregnancy and T1D in childhood.

Exposure		T1D cases	Population size	Algorithm 1^*^: Prescription claims and hospitalisation	
				HR (95% CI) for T1D unadjusted	HR^a^ (95% CI) for T1D, adjusted for all variables
Antibiotics courses (continuous variable)		1,131	315,789	1.06 (1.03–1.08)	1.05 (1.03–1.08)
Antibiotics during pregnancy	No	777	222,099	Ref	Ref
	Yes	354	93,687	1.18 (1.04–1.33)	1.20 (1.05–1.38)
Antibiotics courses during pregnancy	0	780	222,102	Ref	Ref
	1	219	59,361	1.13 (0.97–1.32)	1.17 (1.00–1.37)
	2	66	21,438	0.96 (0.74–1.23)	1.02 (0.78–1.32)
	≥ 3	72	12,888	1.73 (1.36–2.21)	1.85 (1.44–2.38)
Test for trend					0.0008
Antibiotics during pregnancy by type (class), ever user vs never any	Penicillin	282	76,413	1.13 (0.99–1.30)	1.16 (1.01–1.34)
	Other antibiotics	21	5,316	1.06 (0.67–1.67)	1.00 (0.62–1.60)
	Macrolides	66	16,476	1.17 (0.91–1.50)	1.18 (0.91–1.53)
	Cephalosporins and cephamycins	48	9,756	1.46 (1.09–1.95)	1.42 (1.05–1.92)
	Tetracyclines	9	1,245	1.85 (0.92–3.71)	1.81 (0.90–3.65)
Antibiotic use by spectrum (ever vs never)	Narrow	84	25,749	0.95 (0.76–1.18)	1.07 (0.85–1.34)
	Moderate	42	8,904	1.49 (1.10–2.03)	1.57 (1.13–2.18)
	Broad	240	60,681	1.22 (1.06 – 1.40)	1.30 (1.12–1.51)
^a^ HRs were adjusted for: sex, ethnicity, NZ deprivation index, rurality, maternal age at delivery, gestational age and mode of obstetric delivery. * Results are presented based on the first algorithm used for identifying T1D cases. See Supplementary Table S2 for detailed descriptions of the algorithms. Note: counts are randomly rounded to comply with Statistics New Zealand’s data privacy requirements; as a result, totals may be slightly different across different variables and analyses.					

Analysis of five different antibiotic classes revealed that the risk of T1D was significant for both penicillin and cephalosporin/cephamycin. Additionally, both moderate and broad-spectrum antibiotics were significantly associated with T1D; no association was found for narrow-spectrum antibiotics.

Additional analyses examining potential effect modification by key covariates (child sex, ethnicity, socioeconomic deprivation, and mode of obstetric delivery) found no evidence of interaction for either prenatal or postnatal antibiotic exposure (Supplementary Table S3), indicating that associations were consistent across subgroups. Also, including a 6-month latency period did not alter the results (Supplementary Table S4).

### Antibiotics during the first 5 years of life

During the first 5 years, 96.3% of children were prescribed antibiotics at least once. The most prescribed class was Penicillin. The median follow-up was 8.5 years from age 5 (range 0–12 years) until diagnosis or the end of the follow-up period. In total, 0.28% (*n* = 906) of children were diagnosed with T1D after age 5 at a mean age of 10.5 years (Range 0–17 years), using algorithm 1 (see Methods and Supplementary Table S2).

When antibiotic use was analysed as a continuous variable, each additional antibiotic prescription was associated with a 2% increase in risk (Table 3). Analyses comparing ever/never antibiotic users confirmed a positive and significant association (HR 1.62, 95% CI 1.00–2.63; Table 3). Using 20^th^ percentile cut-offs, a significant (*p* for trend = 0.0017) and positive dose-response association was observed. Additional analysis using alternative categories (i.e., based on the 25^th^ percentile) showed comparable results (data not shown).

**Table 3:** Associations between antibiotic use in the first 5 years and subsequent T1D after age 5.

Exposure		T1D cases	Population size	Algorithm 1: Prescription claims and hospitalisation	
				HR (95% CI) for T1D unadjusted	HR^a^ (95% CI) for T1D, adjusted for all variables
Antibiotics courses (continuous variable)		906	315,564	1.02 (1.02–1.03)	1.02 (1.02–1.03)
Antibiotic use	No	18	11,718	Ref	Ref
	Yes	891	303,849	1.71 (1.08–2.73)	1.62 (1.00–2.63)
Antibiotic courses	0	18	11,718	Ref	Ref
	1–4	255	96,078	1.54 (0.96–2.49)	1.45 (0.88–2.37)
	5–7	183	63,771	1.68 (1.04–2.73)	1.61 (0.97–2.65)
	8–12	201	69,375	1.69 (1.04–2.73)	1.60 (0.97–2.63)
	≥ 13	255	74,625	1.99 (1.23–3.21)	1.93 (1.18–3.17)
Test for trend					0.0017
Antibiotic use by type (class), ever user vs never any	Penicillin	876	297,540	1.72 (1.08–2.75)	1.63 (1.01–2.64)
	Other antibiotics	363	113,175	1.86 (1.16–2.99)	1.72 (1.05–2.81)
	Macrolides	336	106,341	1.88 (1.17–3.02)	1.86 (1.14–3.04)
	Cephalosporins and cephamycins	300	94,869	1.92 (1.19–3.09)	1.83 (1.12–3.00)
	Tetracyclines	S	S	S	S
Antibiotic use by type (class), only user vs never any	Penicillin	261	104,520	1.44 (0.89–2.32)	1.36 (0.83–2.23)
	Other antibiotics	S	S	S	S
	Macrolides	9	1,896	2.30 (0.96–5.50)	1.55 (0.27–4.24)
	Cephalosporins and cephamycins	S	S	S	S
	Tetracyclines	S	S	S	S
Antibiotic use by spectrum (ever vs never any)	Narrow	675	226,215	1.15 (0.99–1.33)	1.17 (1.01–1.38)
	Moderate	300	94,863	1.22 (1.06–1.40)	1.23 (1.07–1.42)
	Broad	888	301,995	1.72 (1.11–2.65)	1.74 (1.10–2.78)
Antibiotics (counts) stratified by mode of delivery	Caesarean section (elective)	126	33,027	1.03 (1.01–1.04)	1.02 (1.01–1.04)
	Caesarean section (emergency)	123	42,611	1.02 (1.00–1.03)	1.01 (0.99–1.03)
	Non-caesarean section	639	229,952	1.02 (1.02–1.03)	1.03 (1.02–1.03)
^a^ HRs were adjusted for all variables: sex, ethnicity, NZ deprivation index, rurality, maternal age at delivery, gestational age, mode of obstetric delivery, and birth weight. *Results are presented based on the first algorithm used for identifying T1D cases. See Supplementary Table S2 for detailed descriptions of the algorithms. S: All counts under six and the HRs derived from these are suppressed according to IDI confidentiality requirements. Note: Counts are randomly rounded to comply with Statistics New Zealand’s data privacy requirements; as a result, totals may be slightly different across different variables and analyses.					

When comparing ever users of a specific antibiotic class with those who never used antibiotics, we found significant associations for all classes except tetracyclines, for which the number of prescriptions was too small (Table 3). Since those classified as ever users for a particular class often also used other classes of antibiotics, we also compared individuals who exclusively used one class to those who never used antibiotics. The HRs for these associations were lower, although not statistically significant (Table 3). Analysis by antibiotic spectrum showed significant associations for all antibiotics (narrow, moderate, and broad-spectrum antibiotics), although a slightly weaker association for the narrow-spectrum antibiotics was observed.

When analyses were stratified by mode of obstetric delivery with antibiotic exposure defined as a continuous variable, associations with T1D were statistically significant for both elective caesarean (HR 1.02 95% CI 1.01–1.04) and non-caesarean deliveries (HR 1.03, 95% CI 1.02–1.03), but after adjustment, the association was no longer statistically significant in those born via emergency caesarean delivery (Table 3). No large differences were observed between caesarean and vaginal births.

Similar to analyses involving prenatal exposures, no indication of effect modification by key covariates was observed (Supplementary Table S3); also, including a 6-month latency period did not alter the results (Supplementary Table S4).

### Antibiotic exposure from birth to the second (0–2) year of life

To assess whether the first two years of life were particularly critical for antibiotic exposure and T1D risk, we repeated the analyses using antibiotic exposure in the first two years, with T1D diagnosed after 2 years. These analyses (Supplementary Table S5) showed weaker associations than when using antibiotic consumption in the first 5 years (Table 3).

### Associations stratified by different follow-up periods

For both pre- and post-natal exposures, stratification by different follow-up periods showed highly similar HRs compared to those reported in Tables 2 and 3 and continued to show an elevated risk of T1D (associations for stratified analyses were no longer statistically significant due to reduced power resulting from fewer events within the restricted follow-up windows; Supplementary Table S6).

### Different T1D definitions

Using algorithms 2 and 3 (see Methods and Supplementary Table S2), we identified slightly fewer cases than with algorithm 1 (prenatal: 938 and 1,053; 0–5 years: 747 and 909). Despite the differences in case numbers, the results were similar across all three algorithms (data not shown).

## Discussion

This study showed that antibiotic use during both the prenatal and postnatal periods was positively associated with T1D in a dose-dependent fashion and independent of follow-up duration. During the prenatal period, the clearest associations were found for two classes of antibiotics (penicillin and cephalosporins/cephamycins), while for the postnatal period, all classes, except tetracyclines, were associated with T1D. Associations were strongest for moderate and broad-spectrum antibiotics during both periods. Stratified analyses by mode of obstetric delivery showed highly comparable results, suggesting that caesarean section does not affect associations between antibiotics and T1D.

### Prenatal antibiotic exposure

Our finding that prenatal antibiotic exposure was associated with T1D is consistent with results of an earlier study^[16]^, although no evidence of a positive association was found in several others^[11−14]^, including a large Norwegian cohort study^[11]^. The reason for these mixed results is unclear, but it is unlikely due to differences in antibiotic prescribing, as antibiotic-use categories applied between studies to assess dose-response associations were highly comparable between our New Zealand study, characterised by relatively high antibiotic prescribing, and studies conducted in northern Europe, including Norway, which has relatively low antibiotic prescribing.

We found the strongest associations with prenatal penicillin and cephalosporins/cephamycins, which may have stronger effects on specific bacterial groups important for immune system development or metabolic regulation (see Discussion on potential mechanisms below). For example, some genera believed to have immune benefits, e.g., *Bifidobacterium *spp., *Lactobacillus *spp*.*, and *Lactococcus *spp., are particularly sensitive to penicillin^[13]^. However, if that hypothesis were true, we would have expected similar results for postnatal antibiotic use, which was not the case. Also, these same groups of bacteria are sensitive to macrolides, which were not significantly associated with T1D. Alternatively, as both penicillins and cephalosporins are broad-spectrum antibiotics, they may potentially cause more significant gut microbiome dysbiosis, which is consistent with our finding that the strongest associations were found for moderate and broad-spectrum antibiotics, whereas no significant association was observed for narrow-spectrum antibiotics. Another reason for these differences may simply be a lack of power for some antibiotic classes that are less commonly prescribed. Two additional studies have assessed prenatal antibiotic use by class, with mixed findings. While a Swedish study reported no evidence of an association for any of the antibiotic classes examined^[16]^, a recent Finnish study reported an increased risk of T1D for specific antibiotics, namely sulfonamides and trimethoprim^[14]^.

### Postnatal antibiotic exposure

Consistent with our study, previous studies have reported associations with postnatal antibiotic use^[16,40]^, but these often focused on antibiotic use in the first two years of life, based on the premise that the gut microbiome is more vulnerable during that time and the child is more susceptible to developing chronic conditions. Interestingly, in our study, we found the strongest associations for cumulative exposure over the 0–5-year period compared to exposure in the first two years, suggesting that effects may not be limited to infancy. Alternatively, the reason for weaker associations for antibiotic use in the first two years may be because data quality for pharmaceutical data in the IDI was lower in 2005 and 2006 compared to later years (resulting in some misclassification of both antibiotic use and case ascertainment). However, sensitivity analyses excluding these years did not affect the risk estimates (data not shown), suggesting that data quality did not contribute to these differences. Our findings contrast with those of several recent large-scale registry studies from Finland, Denmark, and the United Kingdom, which reported no overall association between antibiotic use and T1D risk^[14,41,42]^. These differences may reflect New Zealand's substantially higher antibiotic consumption rates among OECD countries, differences in exposure windows, antibiotic spectrum profiles, or the longer follow-up to T1D diagnosis in our cohort.

We observed clear dose-response patterns, similar to some previous studies^[13,40]^ that reported a positive association at higher doses of antibiotic use. However, others^[17]^ observed no dose-response association. The stronger association with more frequent antibiotic use might be because of longer-lasting and more profound gut microbiome dysbiosis, with less frequent antibiotic use likely resulting in a quicker recovery of the gut microbiome^[43]^.

Analysis by spectrum showed an increased risk of T1D for all spectra of antibiotics, but it was strongest for broad-spectrum antibiotics, possibly due to their ability to indiscriminately affect both harmful and beneficial bacteria, with resultant effects on the development and regulation of the immune system (see below;^[23,44]^). Two previous studies have examined associations by spectrum^[15,45]^, with only the earlier study finding similar results for broad-spectrum antibiotics. As we were not able to analyse the risk of T1D for exclusive exposure to broad-, medium-, or narrow-spectrum antibiotics (most children were prescribed a range of antibiotics during the 5-year period), we cannot exclude the possibility that some associations were due to confounding.

As maternal T1D is one of the strongest established risk factors for childhood T1D and may also influence antibiotic prescribing in pregnancy, we conducted additional analyses (for both postnatal and prenatal antibiotic use) adjusting for maternal T1D. These showed highly comparable results, suggesting that maternal T1D was not a confounder in our analyses (data not shown).

### Potential mechanisms

Among pregnant mothers, antibiotics may delay or prevent the transmission of beneficial microflora to the child, thus affecting the child’s gut microbiota, potentially increasing susceptibility to adverse health conditions, including T1D^[13]^. Postnatal antibiotic use, and particularly repeat use, may lead, more directly, to an imbalance of beneficial and harmful bacteria^[46]^, with potential downstream effects on immune system development, homeostasis, and subsequent risk of developing immune or autoimmune-related conditions such as T1D^[47]^. In addition, the gut microbiota is involved in various metabolic processes, including the production of short-chain fatty acids that have anti-inflammatory properties; dysbiosis due to antibiotic use can disrupt this process, potentially contributing to inflammation, immune dysregulation, and autoimmunity, which may affect insulin-producing pancreatic beta cells^[48]^, thus increasing the risk of T1D.

Alternatively, associations may not be causal and could be due to reverse causation, with early stages of T1D (pre-diagnosis) resulting in higher antibiotic use. In particular, children with undiagnosed early-stage T1D might be more susceptible to infections, or their symptoms may be mistaken for an infection, both resulting in higher prescription rates. Although we only included T1D cases that were diagnosed after the antibiotic exposure period, this does not completely mitigate this issue, as a diagnosis may not happen until symptoms are sufficiently clear. It is also possible that results were affected by confounding by indication. In particular, if antibiotics were prescribed for infections that might also increase the risk of T1D, the observed association could partly reflect the impact of the underlying infections rather than the antibiotics themselves. Previous cohort studies examining associations between early-life infections (bacterial and viral) and T1D have shown mixed results, with some reporting positive associations^[49]^ and others finding no evidence of an association^[11]^, so it remains unclear whether this type of bias is likely to explain associations with antibiotic use.

### Generalizability across ethnic groups

Stratified analyses by ethnicity showed no significant interaction (*p* > 0.05), suggesting consistent associations across Māori, Pacific, Asian, and NZ European/Other children despite differing T1D incidence and prescribing patterns. Whether results are generalisable across other ethnic groups internationally remains unclear. It is possible that, while the underlying biological mechanisms will likely be the same across populations, the magnitude of the effect of antibiotics may vary, potentially due to differences in baseline gut microbiota composition.

### Strengths and limitations

The key strength of this study is the large population size and the use of linked data, which reduces the risk of recall bias, as it does not rely on self-reported antibiotic use. Secondly, we have used three different algorithms (see Supplementary Table S2) to define T1D, which produced results that were highly comparable, suggesting that the results are robust and did not hinge on a specific criterion included in any of the algorithms used. Similarly, we used different antibiotic exposure metrics with results being consistent across these different categorisations, thus further highlighting the robustness of our findings. Finally, our study is novel in that it is one of only a few studies conducted in a high antibiotic use setting, with associations identified for antibiotic use both *in utero* and in the first 5 years of life.

This study also has limitations. The retrospective cohort design based on administrative data focuses on associations, with limited ability to assess causal inference. Also, the potential of reverse causation and confounding by indication, as discussed above, cannot be excluded. The latter could have been addressed, at least in part, if the reasons for antibiotic prescriptions were available, but this is not captured in the administrative data currently available. Another limitation is that the data do not capture antibiotic use during hospitalisations, including those administered during caesarean deliveries, a limitation shared with other linkage studies. Furthermore, while antibiotics can only be claimed with a prescription, we cannot be certain that they were consumed, as our data only capture dispensed prescriptions, not actual usage. In addition, we ran multiple Cox regression models to explore associations between early life antibiotic exposure and childhood T1D without formal adjustment for multiple testing. As a result, some observed associations may represent chance findings (Type I error). Further, residual confounding from shared familial genetic and environmental factors may remain despite adjustment for a range of covariates. Finally, as noted above, we were not able to analyse the risk of T1D for exclusive exposure to broad-, medium-, or narrow-spectrum antibiotics. Future studies could further examine dose-response gradients within specific antibiotic-exposed subgroups, which may help disentangle the effects of specific antibiotic subclasses.

## Conclusions

In conclusion, our study suggests that antibiotic use during pregnancy and in the first 5 years of life is associated with an increased risk of developing childhood T1D. This finding further highlights the need for improved antibiotic stewardship, particularly in countries with high prescribing rates like New Zealand^[50]^. Further research would also be valuable to confirm the causal nature of this association, the likely mechanism, and how this effect can be modified in addition to reducing exposure to antibiotics.

## SUPPLEMENTARY DATA

Supplementary data to this article can be found online.

## Data Availability

The data used in this study were provided by Statistics New Zealand, which manages the Integrated Data Infrastructure (IDI), a database combining administrative data from multiple government agencies. These data were originally collected by various New Zealand government ministries and agencies for administrative and statistical purposes. Access to the data for this study was granted by Statistics New Zealand under strict confidentiality and security protocols.
