# Supplementary material for: Early life antibiotic-use and risk of childhood type 1 diabetes: a nationwide cohort study utilising data linkage
Source: Pediatr Diabetes. 2026 Jun 24;2026:e004. doi: 10.48130/pedi-0026-0003 (PMC13463375; doi:10.48130/pedi-0026-0003)
Supplement: Supplementary file 1 [file pedi-2026-2026-1-0003-S1.zip › 10.48130_pedi-0026-0003-Suppl-TableS1.pdf]

Supplementary Table S1. Antibiotics by class and spectrum of activity (Source: Drugs & Medications A to Z – Drugs.com).

Class of antibiotics	Chemical name of antibiotic used among the cohort	Spectrum of activity (Narrow/ Broad)	Antibiotics targets Gram +ve / Gram -ve bacteria
Cephalosporins and Cephamycins	Cefaclor monohydrate	Moderate	Both
	Cefalexin	Moderate	Both
	Cefamandole nafate	Broad	Both
	Cefazolin	Moderate	Both
	Cefoxitin sodium	Moderate	Both
	Ceftazidime	Broad	Both
	Ceftriaxone	Broad	Both
	Cefuroxime axetil	Moderate	Both
	Cefuroxime sodium	Moderate	Both
	Cephalothin sodium	Broad	Both
	Cephradine	Broad	Both
Macrolides	Azithromycin	Broad	Positive
	Clarithromycin	Broad	Positive
	Erythromycin	Broad	Positive
	Erythromycin (as lactobionate)	Broad	Positive
	Erythromycin estolate	Broad	Positive
	Erythromycin ethyl succinate	Narrow	Positive
	Erythromycin stearate	Narrow	Positive
	Roxithromycin	Broad	Positive
Other Antibiotics	Aztreonam	Broad	Negative
	Chloramphenicol	Broad	Both
	Chloramphenicol sodium succinate	Broad	Both
	Ciprofloxacin	Broad	Both
	Clindamycin	Broad	Both
	Colistin sulphomethate	Broad	Positive
	Fleroxacin	Broad	Both
	Framycetin sulphate	Broad	Both
	Gentamicin sulphate	Broad	Both
	Imipenem	Broad	Both
	Levofloxacin	Broad	Both
	Lincomycin	Broad	Both
	Lincomycin hydrochloride	Narrow	Positive
	Moxifloxacin	Broad	Both
	Neomycin sulphate	Broad	Both
	Ofloxacin	Broad	Both
	Paromomycin	Broad	Positive
	Pyrimethamine	Broad	Both
	Sodium Fusidate [fusidic acid]	Narrow	Both
	Spectinomycin hydrochloride	Moderate	Both
	Spiramycin	Broad	Both
	Sulfadiazine sodium	Broad	Both
	Sulphadiazine	Broad	Both
	Tobramycin	Broad	Both
	Triacetyloleandomycin	Broad	Positive
	Trimethoprim	Broad	Both
	Trimethoprim with sulphamethoxazole [Co-trimoxazole]	Broad	Both
	Vancomycin	Narrow	Positive
Penicillins	Amoxicillin	Broad	Both
	Amoxicillin with clavulanic acid	Broad	Both
	Amoxycillin Clavulanate	Broad	Both
	Benzathine benzylpenicillin	Narrow	Both
	Benzylpenicillin sodium [Penicillin G]	Narrow	Both
	Dicloxacillin	Narrow	Both
	Flucloxacillin	Narrow	Both
	Flucloxacillin magnesium	Narrow	Both
	Penicillin G benzathine [Benzathine benzylpenicillin]	Narrow	Both
	Phenoxymethylpenicillin (Penicillin V)	Narrow	Both
	Piperacillin	Broad	Both
	Pivampicillin	Broad	Both
	Pivmecillinam Hydrochloride	Narrow	Both
	Procaine penicillin	Narrow	Both
	Ticarcillin	Broad	Both
Tetracyclines	Demeclocycline hydrochloride	Broad	Both
	Doxycycline	Broad	Both
	Lymecycline	Broad	Both
	Minocycline hydrochloride	Broad	Both
	Rolitetracycline	Broad	Both
	Tetracycline	Broad	Both
	Tetracycline hydrochloride	Broad	Both
