# Supplementary material for: Early life antibiotic-use and risk of childhood type 1 diabetes: a nationwide cohort study utilising data linkage
Source: Pediatr Diabetes. 2026 Jun 24;2026:e004. doi: 10.48130/pedi-0026-0003 (PMC13463375; doi:10.48130/pedi-0026-0003)
Supplement: Supplementary file 1 [file pedi-2026-2026-1-0003-S1.zip › 10.48130_pedi-0026-0003-Suppl-TableS2.pdf]

Supplementary Table 2. Type 1 diabetes ICD-10 codes, description of codes and case definition using 3 different algorithms

ICD-10 Codes-8th Edition	ICD-10 Codes Description (ICD-10 Australian Modification)	Case Definitions
E100	Type 1 diabetes mellitus with hyperosmolarity	Three different algorithms are used to identify cases of T1D:<br><br>Algorithm 1: An individual is identified as a case if they meet one of the following criteria: 1) hospital discharge with one or more ICD-10 codes as outlined in this table; OR 2) prescription of an insulin medication during the study period.<br><br>Algorithm 2: A base dataset for all possible indications of diabetes (i.e. Type 1 and Type 2 diabetes) is extracted first from the cohort dataset. Further processing is based on a previously defined algorithm (McKergow et al., 2017), which identifies an individual as a case if they meet all of the following criteria during the study period: 1) prescription of an insulin medication; AND 2) not dispensed oral hypoglycaemics or alpha glucosidase inhibitors; AND 3) hospital discharges without a T2D diagnosis and at least one hospital discharge with a T1D diagnosis; AND 4) did not die during the study period with a T2D diagnosis in death records; AND 5) no hospital discharges with cystic fibrosis, pancreatectomy, or neonatal diabetes mellitus before insulin dispensing.<br><br>Algorithm 3: A base dataset for all possible indications of diabetes (i.e. Type 1 and Type 2 diabetes) is extracted first from the cohort dataset. Further processing is based on a previously identified algorithm in "Health Tracker", that was developed by the NZ Ministry of Health (Ministry of Health, 2016), which identifies an individual as a T1D case if they meet one of the following criteria during the study period: 1) one or more attendances for NNPAC services under T1D purchase unit codes; OR 2) discharge/diagnosis events/prescriptions related to T1D in the National Minimum Dataset (NMDS)/ Mental Health Information National Collection (MHINC)/ Programme for Integration of Mental Health Data (PRIMHD) /Pharmaceutical Collection (PHARMS). Individuals can also be identified as having T1D based on having discharge or other diagnosis events related to T1D and T2D, along with specific prescription patterns, such as no oral hypoglycemic/metformin prescription and one or more insulin prescriptions. Age-specific criteria further refine the classification for individuals aged 0-14 years or 15 years and older based on the distribution of T1D and T2D events.<br><br>The year of diagnosis for T1D will be based on the date of the first insulin prescription or hospital admission for T1D.
E101	Type 1 diabetes mellitus with acidosis	
E102	Type 1 diabetes mellitus with kidney complication	
E103	Type 1 diabetes mellitus with ophthalmic complication	
E104	Type 1 diabetes mellitus with neurological complication	
E105	Type 1 diabetes mellitus with circulatory complication	
E106	Type 1 diabetes mellitus with other specified complication	
E107	Type 1 diabetes mellitus with multiple complications	
E108	Type 1 diabetes mellitus with unspecified complication	
E109	Type 1 diabetes mellitus without complication	
