# Supplementary material for: Early life antibiotic-use and risk of childhood type 1 diabetes: a nationwide cohort study utilising data linkage
Source: Pediatr Diabetes. 2026 Jun 24;2026:e004. doi: 10.48130/pedi-0026-0003 (PMC13463375; doi:10.48130/pedi-0026-0003)
Supplement: Supplementary file 1 [file pedi-2026-2026-1-0003-S1.zip › 10.48130_pedi-0026-0003-Suppl-TableS3.pdf]

Supplementary Table S3. Stratified analysis by selected covariates for associations between pre and postnatal (first five years) antibiotic exposure (yes vs no) and T1D.

				ALGORITHM 1[*]: Prescription Claims & Hospitalisation	
Exposure period/ Effect modifier	Population sub-groups	T1D Cases	Population Size	HR[a] (95% CI) for T1D adjusted for all variables	p-for interaction
Prenatal					
Sex	Male	552	162,567	1.26 (1.04-1.52)	0.9912
	Female	582	153,222	1.24 (1.03-1.49)	
Ethnicity	NZ Europeans	663	144,306	1.27 (1.07-1.52)	0.9894
	Asian	48	30,543	1.50 (0.78-2.86)	
	MELAA	12	3,816	1.19 (0.37-3.87)	
	Māori	291	98,544	1.20 (0.94-1.53)	
	Others	18	4,560	1.11 (0.36-3.42)	
	Pacific	102	34,017	1.18 (0.79-1.77)	
Deprivation index quintile	1 (Least deprived)	150	38,643	1.17 (0.80-1.73)	0.8611
	2	189	47,820	1.32 (0.95-1.83)	
	3	222	54,423	1.41 (1.06-1.88)	
	4	225	63,285	1.21 (0.91-1.60)	
	5 (Most deprived)	282	89,016	1.16 (0.91-1.48)	
Delivery category	Non-Caesarean Section	801	230,115	1.24 (1.06-1.45)	0.8551
	Caesarean Section (Elective)	150	33,051	1.40 (0.99-1.97)	
	Caesarean Section (Emergency)	156	42,645	1.18 (0.83-1.68)	
	Missing	21	9,510	0.96 (0.35-2.67)	
Postnatal (first 5 years)					
Sex	Male	435	162,378	1.51 (0.75-3.04)	0.7684
	Female	471	153,033	1.75 (0.90-3.38)	
Ethnicity	NZ Europeans	513	144,126	1.67 (0.92-3.04)	0.9643
	Asian	39	30,495	0.78 (0.19-3.26)	
	MELAA	12	3,804	NE[*]	
	Māori	246	98,484	1.69 (0.54-5.28)	
	Others	12	4,554	NE	
	Pacific	93	33,948	1.70 (0.24-12.27)	
Deprivation index quintile	1 (Least deprived)	123	38,586	5.02 (0.70-35.90)	0.7194
	2	153	47,766	1.88 (0.60-5.90)	
	3	171	54,357	1.56 (0.58-4.20)	
	4	177	63,213	1.11 (0.46-2.71)	
	5 (Most deprived)	234	88,923	1.33 (0.50-3.59)	
Delivery category	Non-Caesarean Section	639	229,950	1.57 (0.89-2.79)	0.9335
	Caesarean Section (Elective)	126	33,027	1.79 (0.44-7.24)	
	Caesarean Section (Emergency)	123	42,612	1.07 (0.34-3.36)	
	Missing	18	9,354	NE	

NE = Not estimable (Estimates for some ethnic subgroups were not estimable due to small cell counts and sparse outcome data, resulting in unstable hazard ratio estimates).
[a] *HRs were adjusted for sex, ethnicity, NZ deprivation index, rurality, maternal age at delivery, gestational age, mode of obstetric delivery, and birth weight (only for postnatal exposure model).*
**Results are presented based on the first algorithm used for identifying T1D cases. See Supplementary Table 2 for detailed descriptions of the algorithms.*
S: *All counts under six and the HRs derived from these are suppressed according to IDI confidentiality requirements.*
