# Supplementary material for: Early life antibiotic-use and risk of childhood type 1 diabetes: a nationwide cohort study utilising data linkage
Source: Pediatr Diabetes. 2026 Jun 24;2026:e004. doi: 10.48130/pedi-0026-0003 (PMC13463375; doi:10.48130/pedi-0026-0003)
Supplement: Supplementary file 1 [file pedi-2026-2026-1-0003-S1.zip › 10.48130_pedi-0026-0003-Suppl-TableS4.pdf]

Supplementary Table S4. Associations between antibiotic-use and T1D excluding cases diagnoses within 6 months of follow-up.

			ALGORITHM 1*: Prescription Claims & Hospitalisation	
Exposure variable	T1D cases	Population size	HR (95% CI) for T1D unadjusted	HR[a] (95% CI) for T1D, adjusted for all variables
PRENATAL				
Antibiotics courses (continuous variable)	1119	315,774	1.06 (1.03-1.08)	1.05 (1.03-1.08)
Antibiotic-use (Yes Vs No)				
No	768	222,090	Ref	Ref
Yes	351	93,684	1.17 (1.03-1.33)	1.23 (1.08-1.41)
Antibiotic courses				
0	771	222,093	Ref	Ref
1	219	59,361	1.14 (0.98-1.32)	1.18 (1.00-1.38)
2	66	21,438	0.95 (0.74-1.23)	1.02 (0.78-1.32)
≥3	69	12,885	1.70 (1.33-2.18)	1.86 (1.44-2.39)
Test for trend				0.0013
Antibiotic-use by type (class), ever user VS never any				
Penicillin	279	76,410	1.13 (0.99-1.29)	1.16 (1.01-1.34)
Other antibiotics	18	5,319	1.07 (0.68-1.69)	1.02 (0.64-1.63)
Macrolides	66	16,476	1.18 (0.92-1.52)	1.20 (0.93-1.56)
Cephalosporins & cephamycins	48	9,753	1.48 (1.10-1.97)	1.44 (1.06-1.95)
Tetracyclines	9	1,248	1.87 (0.93-3.75)	1.64 (0.78-3.45)
Antibiotic-use by spectrum (ever vs never)				
Narrow	81	25,746	0.95 (0.76-1.18)	1.06 (0.84-1.33)
Moderate	42	8,901	1.47 (1.08-2.01)	1.55 (1.11-2.16)
Broad	240	60,681	1.22 (1.06-1.41)	1.32 (1.13-1.53)
POSTNATAL (first 5 years)				
Antibiotics courses (continuous variable)	885	314,814	1.02 (1.02-1.03)	1.02 (1.02-1.03)
Antibiotic-use (Yes Vs No)				
No	18	11,634	Ref	Ref
Yes	867	303,180	1.66 (1.04-2.65)	1.57 (0.97-2.53)
Antibiotic courses				
0	18	11,634	Ref	Ref
1-4	246	95,832	1.50 (0.93-2.41)	1.40 (0.85-2.29)
5-7	177	63,657	1.63 (1.00-2.65)	1.55 (0.94-2.55)
8-12	195	69,240	1.65 (1.02-2.67)	1.55 (0.94-2.55)
≥13	246	74,451	1.92 (1.19-3.10)	1.86 (1.13-3.05)
Test for trend				0.0022
Antibiotic-use by type (class), ever user VS never any				
Penicillin	852	296,886	1.67 (1.05-2.66)	1.58 (0.97-2.55)
Other antibiotics	354	112,941	1.80 (1.12-2.89)	1.67 (1.02-2.72)
Macrolides	327	106,140	1.82 (1.13-2.92)	1.80 (1.10-2.95)
Cephalosporins & cephamycins	294	94,665	1.86 (1.16-3.00)	1.79 (1.09-2.93)
Tetracyclines	S	S	S	S
Antibiotic-use by type (class), only user VS never any				
Penicillin	252	104,277	1.39 (0.86-2.25)	1.30 (0.79-2.13)
Other antibiotics	S	S	S	S
Macrolides	6	1,896	2.30 (0.96-5.50)	1.55 (0.57-4.24)
Cephalosporins & cephamycins	S	1,737	S	S
Tetracyclines	S	S	S	S
Antibiotic-use by spectrum (ever vs never any)				
Narrow	657	225,74	1.15 (0.99-1.34)	1.18 (1.01-1.38)
Moderate	294	94,659	1.22 (1.06-1.40)	1.24 (1.07-1.43)
Broad	861	301,332	1.67 (1.08-2.57)	1.69 (1.06-2.69)
Antibiotics (counts) stratified by mode of delivery				
Caesarean Section (Elective)	126	32,955	1.02 (1.01-1.04)	1.02 (1.01-1.04)
Caesarean Section (Emergency)	120	42,498	1.01 (1.00-1.03)	1.01 (0.99-1.03)
Non-Caesarean Section	618	229,422	1.02 (1.02-1.03)	1.03 (1.02-1.03)

[a] *HRs were adjusted for: sex, ethnicity, NZ deprivation index, rurality, maternal age at delivery, gestational age, mode of obstetric delivery, and birth weight (only for postnatal exposure model).*

**Results are presented based on the first algorithm used for identifying T1D cases. See Supplementary Table 2 for detailed descriptions of the algorithms.*

Note: Counts are randomly rounded to comply with Statistics New Zealand's data privacy requirements; as a result, totals may be slightly different across different variables and analyses.
