# Supplementary material for: Early life antibiotic-use and risk of childhood type 1 diabetes: a nationwide cohort study utilising data linkage
Source: Pediatr Diabetes. 2026 Jun 24;2026:e004. doi: 10.48130/pedi-0026-0003 (PMC13463375; doi:10.48130/pedi-0026-0003)
Supplement: Supplementary file 1 [file pedi-2026-2026-1-0003-S1.zip › 10.48130_pedi-0026-0003-Suppl-TableS5.pdf]

Supplementary Table S5. Associations between antibiotic-use in the first two years and subsequent T1D after age two years

			ALGORITHM 1*: Prescription Claims & Hospitalisation	
EXPOSURE / EXPOSURE PERIOD	T1D Cases	Population Size	HR (95% CI) for T1D unadjusted	HR[a] (95% CI) for T1D, adjusted for all variable
0-2 YEARS				
Antibiotics (counts)	1071	315,726	1.02 (1.01-1.03)	1.02 (1.01-1.04)
Antibiotics (YES/ NO)				
NO	162	51,786	Ref	Ref
YES	906	263,943	1.07 (0.91-1.27)	1.08 (0.91-1.29)
Antibiotics courses				
0	165	51,786	Ref	Ref
1-2	327	95,691	1.07 (0.88-1.29)	1.06 (0.87-1.28)
3-4	213	64,803	1.02 (0.83-1.25)	1.03 (0.84-1.28)
5-6	135	40,440	1.04 (0.83-1.30)	1.08 (0.85-1.36)
≥7	234	63,009	1.16 (0.95-1.41)	1.20 (0.97-1.48)
Test for trend in HR				0.2669
Antibiotics by type (class), ever user VS never any				
Penicillin	870	252,513	1.08 (0.92-1.28)	1.10 (0.93-1.31)
Other antibiotics	270	66,852	1.24 (1.02-1.50)	1.25 (1.02-1.54)
Macrolides	183	54,615	1.06 (0.86-1.30)	1.12 (0.90-1.40)
Cephalosporins & cephamycins	198	52,560	1.21 (0.98-1.49)	1.21 (0.97-1.51)
Tetracyclines	S	S	S	S
Antibiotics by type (class), only user VS never any				
Penicillin	435	136,308	0.99 (0.83-1.19)	1.00 (0.83-1.21)
Other antibiotics	9	3,528	0.88 (0.47-1.67)	0.76 (0.37-1.55)
Macrolides	12	3,345	1.06 (0.57-1.95)	1.03 (0.54-1.95)
Cephalosporins & cephamycins	9	2,619	0.86 (0.41-1.84)	0.80 (0.35-1.81)
Tetracyclines	S	S	S	S
Antibiotics by type (spectrum), only user VS never any				
Narrow	453	140,271	0.92 (0.82-1.04)	0.95 (0.84-1.08)
Moderate	201	52,554	1.18 (1.01-1.38)	1.18 (1.00-1.38)
Broad	915	265,398	1.09 (0.92-1.29)	1.08 (0.91-1.29)

[a]*HRs were adjusted for all variables: sex, ethnicity, NZ deprivation index, rurality, maternal age at delivery, gestational age, mode of obstetric delivery, and birth weight.*
**Note: Results are presented based on the first algorithm used for identifying T1D cases. See the supplementary table for detailed descriptions of the algorithms.*
S: *All counts under six and the HRs derived from these are suppressed according to IDI confidentiality requirements.*
Note: Counts are randomly rounded to comply with Statistics New Zealand's data privacy requirements; as a result, totals may be slightly different across different variables and analyses.
