# Supplementary material for: Early life antibiotic-use and risk of childhood type 1 diabetes: a nationwide cohort study utilising data linkage
Source: Pediatr Diabetes. 2026 Jun 24;2026:e004. doi: 10.48130/pedi-0026-0003 (PMC13463375; doi:10.48130/pedi-0026-0003)
Supplement: Supplementary file 1 [file pedi-2026-2026-1-0003-S1.zip › 10.48130_pedi-0026-0003-Suppl-TableS6.pdf]

Supplementary Table S6. Association between antibiotic exposure and T1D by follow-up duration

			ALGORITHM 1*: Prescription Claims & Hospitalisation	
Follow-up duration from exposure widow	T1D cases	Population size	HR (95% CI) for T1D unadjusted	HR[a] (95% CI) for T1D, adjusted for all variables
Prenatal exposure				
0-5 years	222	315,786	1.14 (0.86-1.51)	1.23 (0.91-1.65)
≥5-10 years	387	315,564	1.16 (0.94-1.44)	1.24 (0.99-1.54)
≥10 years	519	315,177	1.18 (0.98-1.43)	1.19 (0.97-1.45)
Postnatal exposure (age 0-5 years)				
0-5 years	390	315,564	1.84 (0.91-3.70)	1.74 (0.86-3.51)
>5 years	519	315,177	1.95 (1.05-3.65)	1.85 (0.96-3.59)

[a] *HRs were adjusted for: sex, ethnicity, NZ deprivation index, rurality, maternal age at delivery, gestational age, mode of obstetric delivery, and birth weight (only for postnatal exposure model).*
**Results are presented based on the first algorithm used for identifying T1D cases. See Supplementary Table 2 for detailed descriptions of the algorithms.*
Note: Counts are randomly rounded to comply with Statistics New Zealand's data privacy requirements; as a result, totals may be slightly different across different variables and analyses.
