# Supplementary material for: Early life antibiotic-use and risk of childhood type 1 diabetes: a nationwide cohort study utilising data linkage
Source: Pediatr Diabetes. 2026 Jun 24;2026:e004. doi: 10.48130/pedi-0026-0003 (PMC13463375; doi:10.48130/pedi-0026-0003)
Supplement: Supplementary file 1 [file pedi-2026-2026-1-0003-S1.zip › 10.48130_pedi-0026-0003-Suppl-FigureS1.pdf]

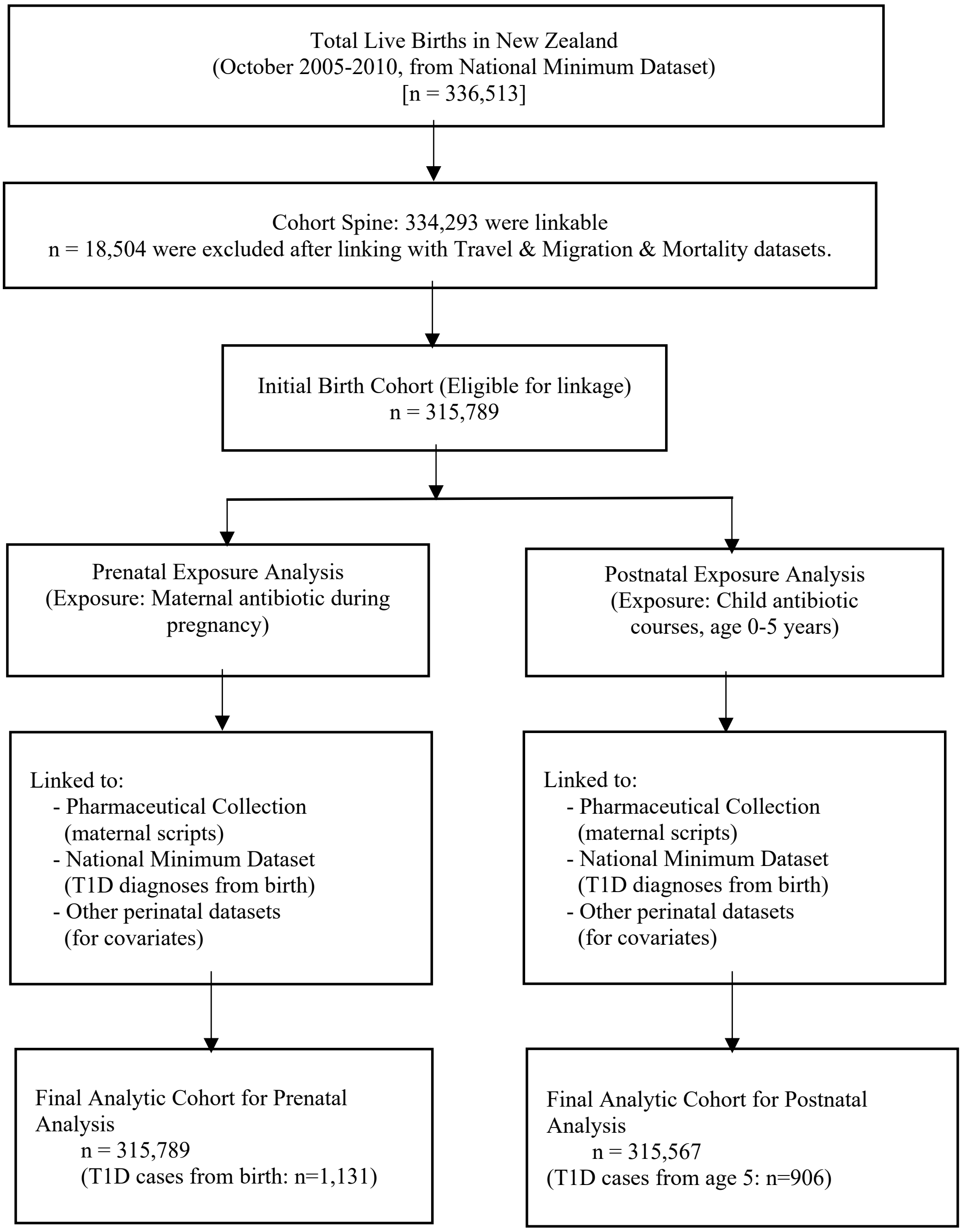

Note: Counts (n) are randomly rounded to comply with Statistics New Zealand's data privacy requirements; as a result, totals may be slightly different across different variables and analyses.
Postnatal analysis cohort has fewer subjects as it excludes any child diagnosed with T1D before their 5th birthday.

Supplementary Figure S1: Cohort derivation flow diagram.
